# Coupled regulations of enzymatic activity and structure formation of aldehyde dehydrogenase Ald4p

**DOI:** 10.1242/bio.051110

**Published:** 2020-04-28

**Authors:** Chalongrat Noree, Naraporn Sirinonthanawech

**Affiliations:** Institute of Molecular Biosciences, Mahidol University, 25/25 Phuttamonthon 4 Road, Salaya, Phuttamonthon, Nakhon Pathom 73170, Thailand

**Keywords:** Aldehyde dehydrogenase, Extramitochondrial assembly, Yeast

## Abstract

Previously, we have developed an extramitochondrial assembly system, where mitochondrial targeting signal (MTS) can be removed from a given mitochondrial enzyme, which could be used to characterize the regulatory factors involved in enzyme assembly/disassembly *in vivo*. Here, we demonstrate that addition of exogenous acetaldehyde can quickly induce the supramolecular assembly of MTS-deleted aldehyde dehydrogenase Ald4p in yeast cytoplasm. Also, by using PCR-based modification of the yeast genome, cytoplasmically targeted Ald4p cannot polymerize into long filaments when key functional amino acid residues are substituted, as shown by N192D, S269A, E290K and C324A mutations. This study has confirmed that extramitochondrial assembly could be a powerful external system for studying mitochondrial enzyme assembly, and its regulatory factors outside the mitochondria. In addition, we propose that mitochondrial enzyme assembly/disassembly is coupled to the regulation of a given mitochondrial enzyme activity.

## Abstract (precis)

**Summary:** A mutation study of cytoplasmically targeted aldehyde dehydrogenase Ald4p was conducted to show that the Ald4p structure formation is coupled to the regulation of its enzymatic activity.

## INTRODUCTION

During alcoholic fermentation, yeast produces excessive amounts of acetaldehyde, a toxic and stress-inducible substance. For this reason, yeast aldehyde dehydrogenases play an important role in lowering the intracellular levels of acetaldehyde by converting it to another metabolically utilizable molecule, acetate [acetaldehyde+NAD(P)^+^+H_2_O→acetate+NAD(P)H]. Aldehyde dehydrogenases, in *Saccharomyces cerevisiae*, are encoded by *ALD2*, *ALD3*, *ALD4*, *ALD5* and *ALD6*, and they can distinctly be found in two subcellular locations. Ald2p, Ald3p and Ald6p are in cytoplasm, whereas Ald4p and Ald5p are targeted to mitochondria. As a result, the expression of mitochondrial aldehyde dehydrogenases is regulated differently from their cytosolic isoenzymes. The presence of glucose can only trigger the expression of cytosolic aldehyde dehydrogenases, but not the expression of their mitochondrial counterparts. In the case of Ald4p, it can be activated in the presence of alcohol and acetaldehyde (Jacobson and Bernofsky, 1974; Wang et al., 1998; Tessier et al., 1998; Navarro-Aviño et al., 1999; Boubekeur et al., 2001; Aranda and del Olmo, 2003; Saint-Prix et al., 2004; Mukhopadhyay et al., 2013).

A previous study has revealed that Ald4p is a component of needle-like structures found in mitochondria, suggesting the potential role of Ald4p in supramolecular assembly (Misonou et al., 2014). Since the mitochondrion is such a small compartment, our recent work has developed an extramitochondrial system by removing mitochondrial targeting sequence (MTS) from Ald4p such that it can successfully be restricted in the cytoplasm. The cytoplasmically targeted Ald4p can also exhibit the supramolecular assembly into long filaments when growing cells to saturation and stationary phase, and its assembly can be reversible when fresh medium is reintroduced (Noree, 2018).

In the studies with filament/punctate-forming cytosolic enzymes, it has been shown that enzyme assembly is coupled to the regulation of their enzyme activity (Petrovska et al., 2014; Barry et al., 2014; Noree et al., 2014; Narayanaswamy et al., 2009; Lynch et al., 2017; An et al., 2008; Noree et al., 2019). However, it is not known yet if the assembly of mitochondrial Ald4p is, in a similar fashion, linked to the control of Ald4p activity. Therefore, in this study we address this question by treating cells with acetaldehyde to monitor if the assembly of MTS-deleted and MTS-intact Ald4p-GFP is associated with the availability of its substrate. Moreover, we introduced some single mutations to the enzyme that can either affect enzyme activity, substrate binding/recognition, or tetrahedral transition state stabilization. We have found that, like cytosolic enzymes capable of self-assembly, Ald4p assembly is utilized by cells to modulate Ald4p activity. Our work has confirmed the usefulness of extramitochondrial assembly as a system for studying regulatory factors involved in high-order structure formation of a given mitochondrial enzyme.

## RESULTS AND DISCUSSION

### Extramitochondrial assembly of Ald4p(noMTS)-GFP can be used as a system for characterizing regulatory factors involved in Ald4p assembly/disassembly

As shown previously by our group, Ald4p still retained its ability to form cytoplasmic structures (foci/filaments) when it was relocated from mitochondria to cytoplasm. This highlights its potential as an external platform that can be utilized for characterizing regulatory factors involved in assembly/disassembly of a given mitochondrial enzyme. The assembly of Ald4p(noMTS)-GFP can be observed in yeast *ALD4(noMTS)::GFP* grown to saturation and stationary phase. Although barely present in log-phase culture, the Ald4p(noMTS)-GFP structure formation can quickly be triggered, within 15 min, by shifting log-phase cells to the old culture medium, isolated from 3-day-old yeast culture. The assembly process can also be reversed. Shifting 3-day-old cultured cells to a fresh medium for 15 min can wipe out Ald4p(noMTS)-GFP structures (Noree, 2018). However, it is not yet known if the assembly of Ald4p is coupled to the regulation of its enzyme activity.

**Fig. 1.**
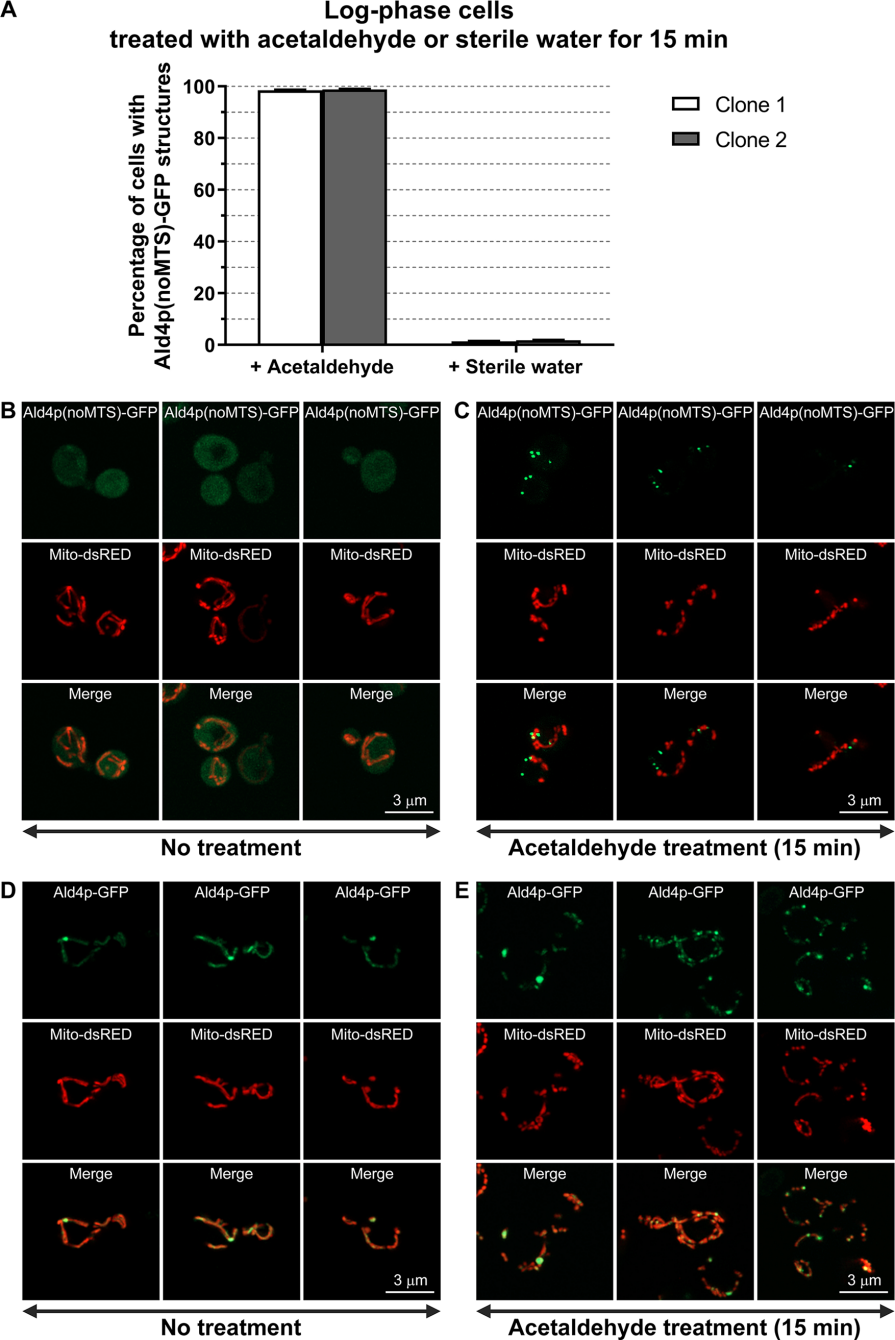
**Ald4p(noMTS)-GFP assembly can quickly be triggered after treatment with exogenous acetaldehyde.** (A) Yeast *ALD4(noMTS)::GFP* transformed with pVTU-mito-dsRED was grown in liquid uracil-dropout medium to log-phase stage of growth at 30°C with shaking. The log-phase cells (1 ml) were then treated either with acetaldehyde (5 µl; 3.9 mg/ml final concentration) or with sterile water (5 µl; as a control), and incubated at 30°C for 15 min with shaking before counting. The cells were then inspected under fluorescence microscope for the percentage of cells with Ald4p(noMTS)-GFP structures. Two different clones were used in the experiments. For each clone, three independent experiments were performed and reported as average±s.e.m. (Raw data and statistical analyses are shown in Table S2). Representative images of yeast *ALD4(noMTS)::GFP* with pVTU-mito-dsRED grown to log-phase, mentioned in A, when treated either with sterile water (B), or with acetaldehyde (C) for 15 min. Representative images of yeast *ALD4(intactMTS)::GFP* with pVTU-mito-dsRED grown to log-phase when treated either with sterile water (D), or with acetaldehyde (E) for 15 min.

To address this question, we first tested whether the structure formation of Ald4p(noMTS)-GFP can be triggered by adding its substrate, acetaldehyde, to the log-phase culture of yeast *ALD4(noMTS)::GFP* (none or very few cells showed structures at this stage of growth). In Fig. 1A to C, a 15-min treatment of log-phase cells with exogenous acetaldehyde can trigger the assembly of Ald4p(noMTS)-GFP to >98%, compared with the control (<2% when treated with sterile water).

To confirm that the assembly induction by acetaldehyde does not only exist for the cytoplasmically targeted Ald4p, the acetaldehyde treatment experiments were also performed with yeast *ALD4(intactMTS)::GFP*. However, the visible structures of Ald4p(intactMTS)-GFP can readily be observed during log-phase, thus making the assembly frequency unchanged when we treated the cells with acetaldehyde. Nonetheless, more structures were obviously present in the acetaldehyde-treated cells than in the non-treated cells, typically carrying only one to three structures per cell (Fig. 1D,E). Altogether, the data from both mitochondrial Ald4p and its cytoplasmically targeted counterpart suggests that the Ald4p assembly associates with the availability of its substrate.

However, we found that the assembly induced by acetaldehyde addition was not caused by changes in pH of the culture medium (Fig. S1).

### Disruption in substrate-enzyme transition state, nucleotide binding site and enzyme active site affected the Ald4p assembly

**Fig. 2.**
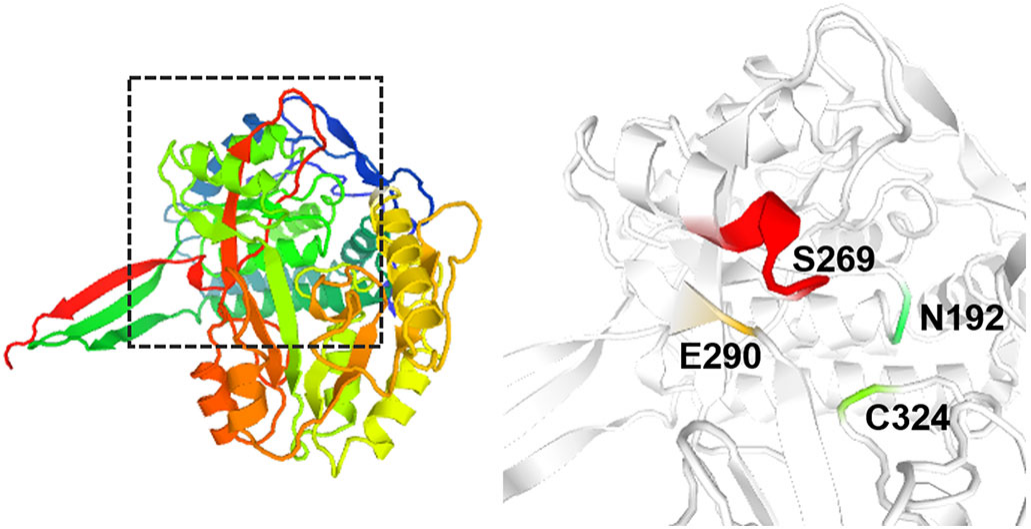
**Three-dimensional** **(3D)** **model of yeast aldehyde dehydrogenase Ald4p and amino acid residues selected for site-directed mutagenesis.** The 3D model of Ald4p (P46367) is provided by MODBASE, based on template ‘1bi9A’ (with 50% sequence identity; alignment shown in Fig. S2; www.proteinmodelportal.org). Four amino acid residues of Ald4p were selected for site-directed mutagenesis in this study; N192 (transition state stabilizer, cyan), S269 (nucleotide binding region, red), E290 (active site; proton acceptor, yellow), and C324 (active site; nucleophile, green). Right panel is a magnification of the dashed box drawn on the left panel (amino acid sequence alignment between yeast Ald4p and human ALDH2 shown in Fig. S3).

Next, we used similar approaches from our previous studies with cytosolic enzymes, CTP synthetase (Noree et al., 2014) and asparagine synthetase (Noree et al., 2019) to investigate whether the assembly and enzyme activity regulation of aldehyde dehydrogenase are coupled events. We decided to introduce some single mutations affecting the Ald4p activity (Fig. 2). By using PCR-based modification of yeast genome technique, we can mutagenize the chromosomal *ALD4*, remove the MTS sequence (encoded by nt1-72) and also introduce the *GFP* after *ALD4* coding sequence, simultaneously.

**Fig. 3.**
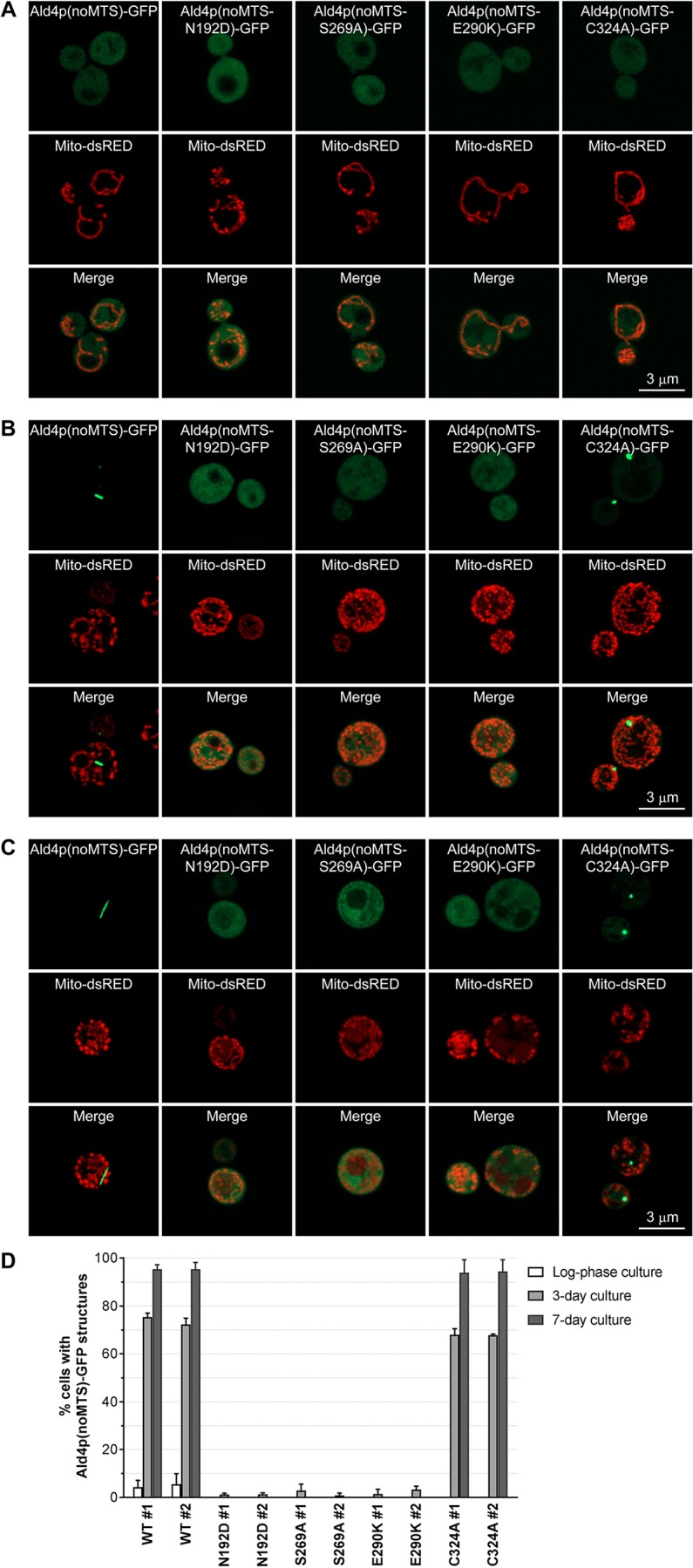
**Ald4p(noMTS)-GFP with N192D, S269A, E290K, or C324A mutation cannot form filaments.** Yeast *ALD4(noMTS)::GFP*, *ALD4(noMTS-N192D)::GFP*, *ALD4(noMTS-S269A)::GFP*, *ALD4(noMTS-E290K)::GFP*, and *ALD4(noMTS-C324A)::GFP*, transformed with pVTU-mito-dsRED, were grown in SC-uracil to log-phase (A), for 3 days (B) and for 7 days (C) at 30°C with shaking. Representative images of live cells were captured in Z-stack for 1–3 µm, and compressed into a single image using maximum projection. (D) Assembly of Ald4p(noMTS)-GFP was inhibited when N192D, S269A, or E290K mutation was present in *ALD4*. For each strain, two different clones were used for analysis. About 250 cells of each clone were counted for each experiment. Three independent experiments were performed and reported as percentage average±s.e.m.

The first single mutation introduced to yeast chromosomal *ALD4* is N192D (equivalent to N186 in human mitochondrial aldehyde dehydrogenase ALDH2). It was selected based on its role in stabilizing the tetrahedral transition state (Steinmetz et al., 1997). When this mutation was incorporated, yeast *ald4(noMTS-N192D)::GFP* showed very low frequency of supramolecular assembly of Ald4p(noMTS-N192D)-GFP in all stages of growth (0% during log phase, <2% at day 3, and 0% at day 7), as shown in Fig. 3 and Table 1. Thus, we conclude that assembly of Ald4p relies on the tetrahedral transition state of enzyme-substrate.

**Table 1.**
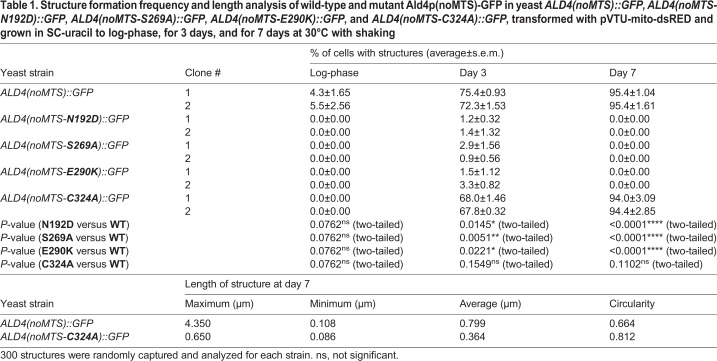
**Structure formation frequency and length analysis of wild-type and mutant Ald4p(noMTS)-GFP in yeast *ALD4(noMTS)::GFP*, *ALD4(noMTS-N192D)::GFP*, *ALD4(noMTS-S269A)::GFP*, *ALD4(noMTS-E290K)::GFP*, and *ALD4(noMTS-C324A)::GFP*, transformed with pVTU-mito-dsRED and grown in SC-uracil to log-phase, for 3 days, and for 7 days at 30°C with shaking**

A key region of NAD(P)^+^ binding, 268-GSTATG-273 (equivalent to 262-GSTEIG-267 in human ALDH2), was previously identified (Liu et al., 1997). Interestingly, S269A mutation also caused a vast reduction of Ald4p(noMTS-S269A)-GFP structure formation (0% during log phase, <3% at day 3, and 0% at day 7) (Fig. 3 and Table 1). This suggests that assembly of Ald4p needs the enzyme being bound with NAD(P)^+^.

Next, we asked if the occupation status of the active site of Ald4p is associated with Ald4p assembly. E290 (equivalent to E285 in human ALDH2) is a residue identified as a proton acceptor (Abriola et al., 1987). When E290K was introduced to the protein, cells with Ald4p(noMTS-E290K)-GFP structures were found to be 0% during log phase, <4% at day 3, and 0% at day 7, of the total cells counted (Fig. 3 and Table 1).

Interestingly, the alanine substitution of nucleophilic residue C324 in the Ald4p active site, equivalent to C319 in human ALDH2 (Hempel and Pietruszko, 1981; Steinmetz et al., 1997), can impair enzyme polymerization into long filaments, showing only foci. We found that, at day 7, the length distribution of the enzyme with C324A mutation was 0.650 µm maximum, 0.086 µm minimum, and 0.364 µm average, significantly different from the wild-type enzyme that showed 4.35 µm maximum, 0.108 µm minimum, and 0.799 µm average length of their structures (Table 1). At this stage of growth, regardless of the shape of the assemblies, yeast *ald4(noMTS-C324A)::GFP* exhibited comparable assembly frequency to yeast *ALD4(noMTS)::GFP* (0% during log phase, about 70% at day 3, and about 95% at day 7) (Fig. 3 and Table 1). It is possible that C324A mutation might not affect enzymatic function but just interfere with the joining of adjacent Ald4p oligomers in polymerizing into long filaments. Further investigations are needed to reveal the C324A mutation effect on polymerization of Ald4p filaments.

Altogether, we demonstrate that extramitochondrial assembly could be used as an external system to identify regulatory factors involved in assembly/disassembly of a given mitochondrial enzyme. In the case of Ald4p, this study has revealed that availability of substrate (acetaldehyde treatment), substrate-enzyme binding and transition state, and enzyme active site are all connected with polymerization of Ald4p into filamentous structures. Consequently, our finding indicates that Ald4p assembly is utilized by cells to modulate enzymatic activity of Ald4p.

## MATERIALS AND METHODS

### Bacteria, yeast strain, growth and selection media

*Escherichia coli* DH5α was used as a host for PCR-based site-directed mutagenesis and plasmid propagation. LB medium [0.5% (w/v) yeast extract (BD Biosciences), 1% (w/v) Bacto-tryptone (BD Biosciences), 1% (w/v) sodium chloride (BDH Prolabo)] supplemented with 100 µg/ml ampicillin (PanReac AppliChem) was used for selection. Bacterial cultures were maintained at 37°C.

Yeast BY4741 (*MATa his3Δ1 leu2Δ0 met15Δ0 ura3Δ0*) was used as a background strain for yeast chromosomal gene modifications. YPD medium [1% (w/v) yeast extract (BD Biosciences), 2% (w/v) Bacto-peptone (BD Biosciences), and 2% (w/v) dextrose (Sigma-Aldrich)] was used for general growth. G418 (PanReac AppliChem) was used for selecting yeast transformants. Uracil-dropout medium (Sigma-Aldrich) ‘SC-uracil’ containing 2% (w/v) dextrose was used to select for yeast transformed with pVTU-mito-dsRED. All yeast constructs were grown at 30°C.

### Acetaldehyde treatment

To study the effect of acetaldehyde, a substrate of Ald4p, on the assembly of Ald4p(noMTS)-GFP and Ald4p(intactMTS)-GFP, two yeast strains *ALD4(noMTS)::GFP* and *ALD4(intactMTS)::GFP*, both transformed with pVTU-mito-dsRED (Noree, 2018), were grown to log-phase in SC-uracil at 30°C with shaking at 250 rpm (Green SSeriker II Model VS-8480FSN, Vision Scientific). 1 ml of log-phase culture was transferred to a microfuge tube. Acetaldehyde (Sigma-Aldrich, 402788, ≥99.5%, density 0.785 g/ml at 25°C) (5 µl) was then added to the tube (about 3.925 mg/ml final concentration). The cells were incubated at 30°C for 15 min with shaking at 300 rpm (ThermoMixer C, Eppendorf). The control was set up similarly except that an equal volume of sterile water (5 µl) was added to the tube, instead.

### PCR-based site-directed mutagenesis

pFA6a-ALD4-GFP-kanMX6, constructed in previous study (Noree, 2018), was used as a DNA template to introduce N192D, S269A, E290K and C324A mutations to the *ALD4* coding sequence within the plasmid. All primers used for mutagenesis are listed in Table S1. Primers were first phosphorylated at their 5′ ends with T4 Polynucleotide Kinase (New England Biolabs). PCR-based site-directed mutagenesis reactions were set up using KOD Hot Start DNA Polymerase (Merck). The plasmid template was removed by *Dpn*I treatment (New England Biolabs). The mutagenized PCR products were purified using GenepHlow™ Gel/PCR Kit (Geneaid). Ligations were set up using T4 DNA Ligase (New England Biolabs) to make mutagenized PCR products (linear plasmids) become circular prior to bacterial transformation. After transformation using heat shock method, plasmids were isolated from the selected transformants using Presto™ Mini Plasmid Kit (Geneaid), and were then verified by DNA sequencing (Macrogen, South Korea). The resulting plasmids after mutagenesis were referred to as pFA6a-ALD4(N192D)-GFP-kanMX6, pFA6a-ALD4(S269A)-GFP-kanMX6, pFA6a-ALD4(E290K)-GFP-kanMX6, and pFA6a-ALD4(C324A)-GFP-kanMX6, respectively.

### Yeast chromosomal gene modification

The information of nucleotide sequence coding for MTS was retrieved from the UniProt database (http://www.uniprot.org/). MTS of *ALD4* is encoded by nt1-72. The 3D model of yeast Ald4p, provided by MODBASE, is predicted based on *Rattus norvegicus* retinal dehydrogenase type two with NAD bound (1bi9A) and retrieved from www.proteinmodelportal.org (model creation and template verification date: January 27, 2017).

PCR-based engineering of the yeast genome (Petracek and Longtine, 2002) was employed to construct the following yeast strains: *ald4(noMTS-N192D)::GFP*, *ald4(noMTS-S269A)::GFP*, *ald4(noMTS-E290K)::GFP*, and *ald4(noMTS-C324A)::GFP*. First, pFA6a-ALD4(N192D)-GFP-kanMX6, pFA6a-ALD4(S269A)-GFP-kanMX6, pFA6a-ALD4(E290K)-GFP-kanMX6, and pFA6a-ALD4(C324A)-GFP-kanMX6, were used as DNA templates for making the DNA cassettes harboring (sequence in order from 5′ to 3′): 50 nt upstream of the *ALD4* start codon, ATG, *ALD4* coding sequence (nt4-72 deleted, in the case of MTS removal, with either N192D, S269A, E290K, or C324A mutation), *GFP*, kanamycin resistance gene, and 50 nt downstream of the *ALD4* stop codon. PCR reactions were set up using the KOD Hot Start DNA Polymerase kit. Yeast BY4741 was transformed with the purified DNA cassettes using lithium acetate/PEG transformation method. YPD supplemented with 400 µg/ml G418 was used for selection. The MTS-deleted and the MTS-intact yeast transformants were initially screened under the fluorescence microscope by checking two distinct localization patterns (cytosolic and mitochondrial, respectively), and were then confirmed by sending out the PCR products of the isolated genomic DNA from the yeast constructs to be verified for DNA sequencing (Macrogen, South Korea). Primers for making the DNA cassettes for yeast transformation and sequencing primers are shown in Table S1.

### Colocalization assay

To confirm the successful relocation of Ald4p(noMTS-N192D)-GFP, Ald4p(noMTS-S269A)-GFP, Ald4p(noMTS-E290K)-GFP, and Ald4p(noMTS-C324A)-GFP, from mitochondria to cytoplasm, yeast *ald4(noMTS-N192D)::GFP*, *ald4(noMTS-S269A)::GFP*, *ald4(noMTS-E290K)::GFP*, and *ald4(noMTS-C324A)::GFP* were transformed with a mitochondrial marker plasmid ‘pVTU-mito-dsRED’ (a gift from J. Wilhelm, UCSD), using lithium acetate/PEG transformation method (500 ng plasmid for each transformation). SC-uracil with 400 µg/ml G418 was used for selecting yeast transformants.

### Monitoring pH values of yeast culture media supplemented with or without acetaldehyde

The culture media from acetaldehyde treatment experiments were isolated after the cell counting had been done. The aliquots were centrifuged at 10,000 rpm for 3 min at room temperature. Only the clear liquid medium was carefully transferred to a new sterile microcentrifuge tube, followed by centrifugation again at 10,000 rpm for 3 min at room temperature. The clear liquid medium from the second round centrifugation was carefully transferred to a new sterile microcentrifuge tube and used to check its pH immediately with pH-indicator strips (Merck). Three independent experiments were performed to confirm the results.

### Cell counting and confocal imaging

Yeast cells were grown to the growth stages or in conditions as indicated. For counting, about 10 µl of cells were put on a microscope slide (Shandon Superfrost Plus, Thermo Fisher Scientific), covered by a coverslip (Menzel Gläser, Thermo Fisher Scientific), and the excess liquid was blotted off before analysis under fluorescence microscope. Cell counting, data presentation, and statistical analyses were performed as described previously (Noree et al., 2014). Briefly, for each strain or each condition tested, cells were randomly counted in five different fields of view, and about 50 cells per field of view (>250 cells in total) were counted in each experiment. Then, the percentage of cells with Ald4p-GFP structures was calculated. The average±s.e.m. of three independent experiments was reported for each condition, and Student's *t*-test was used for statistical analyses (GraphPad Prism 7.03).

For imaging, wet slides were prepared by dropping live cell suspension onto a slide, covering with a coverslip, blotting off excess liquid to prevent cells from floating around, and then sealing edges of the coverslip with nail polish. Images were taken with the Carl Zeiss LSM800 with AiryScan using Plan-Apochromat 63×/1.4 Oil DIC ∞/0.17 objective lens with Zen Blue software version 2.1.57.1000. Quantification of Ald4p(noMTS)-GFP and Ald4p(noMTS-C324A)-GFP structures was performed as described previously (Noree et al., 2014). Briefly, after cell imaging and maximal projection processing, 300 structures of each strain were randomly captured and analyzed for maximum, minimum, average length, and circularity using Zen Blue software version 2.1.57.1000.

## Supplementary Material

Supplementary information
