# Supplementary material for: Coupled regulations of enzymatic activity and structure formation of aldehyde dehydrogenase Ald4p
Source: Biol Open. 2020 Apr 28;9(4):bio051110. doi: 10.1242/bio.051110 (PMC7197708; doi:10.1242/bio.051110)
Supplement: Supplementary information [file biolopen-9-051110-s1.pdf]

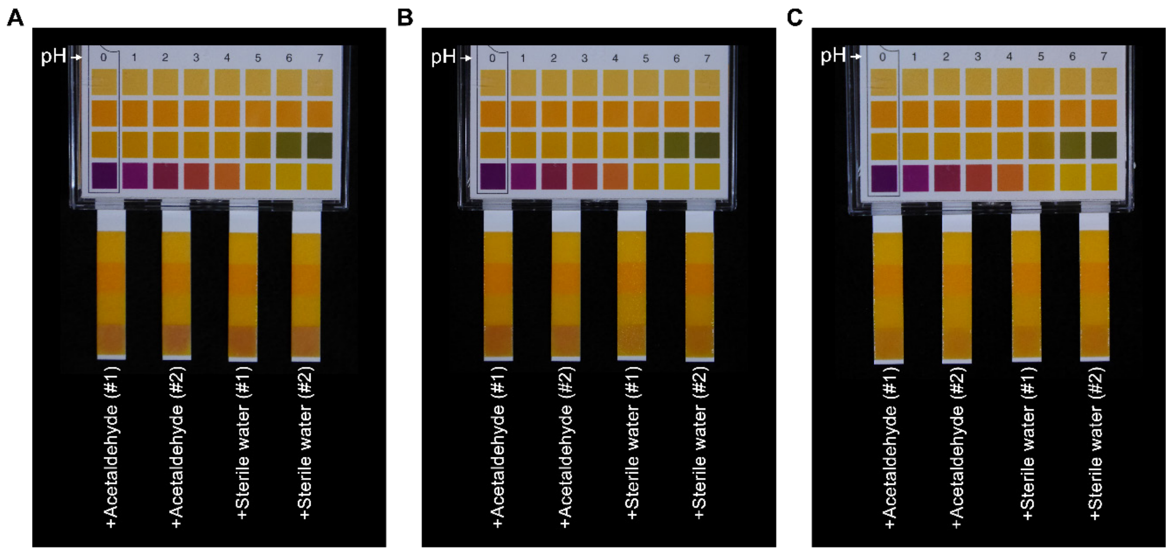

Fig. S1. Assembly induction with exogenous acetaldehyde addition was not triggered by changes in pH of culture medium. Yeast *ALD4(noMTS)::GFP* was grown to log phase. Acetaldehyde (5 µl; 3.9 mg/ml final concentration) or sterile water (5 µl; as a control) was then added to 1 ml of log-phase cells. After incubation for 15 min and counting the cells, the culture media were isolated to monitor their pH. **A-C** show the results of three independent experiments.

Biology Open • Supplementary information

```
Model          ------VPIKLPNGLEYEQPTGLFINNKFVPSKQNKTFEVINPSTEEEICHIYEGREDDV
1bi9A          MASLQLLPS-PTPNLEIK-YTKIFINNEWQNSESGRVFPVCNPATGEQVCEVQEADKVDI
id. residues   .......P......LE....T..FINN....S.....F.V.NP.T.E..C...E....D.

Model          EEAVQAADRAFSN-GSWNGIDPIDRGKALYRLAELIEQDKDVIASIETLDNGK-AISSSR
1bi9A          DKAVQAARLAFSLGSVWRRMDASERGRLLDKLADLVERDRATLATMESLNGGKPFLQAFY
id. residues   ..AVQAA..AFS....W...D...RG..L..LA.L.E.D....A..E.L..GK.......

Model          GDVDLVINYLKSSAGFADKIDGRMIDTGRTHFSYTKRQPLGVCGQIIPWNFPLLMWAWKI
1bi9A          IDLQGVIKTLRYYAGWADKIHGMTIPVDGDYFTFTRHEPIGVCGQIIPWNFPLLMFTWKI
id. residues   .D...VI..L...AG.ADKI.G..I......F..T...P.GVCGQIIPWNFPLLM..WKI

Model          APALVTGNTVVLKTAESTPLSALYVSKYIPQAGIPPGVINIVSGFGKIVGEAITNHPKIK
1bi9A          APALCCGNTVVIKPAEQTPLSALYMGALIKEAGFPPGVVNILPGYGPTAGAAIASHIGID
id. residues   APAL..GNTVV.K.AE.TPLSALY....I..AG.PPGV.NI..G.G...G.AI..H..I.

Model          KVAFTGSTATGRHIY-QSAAAGLKKVTLELGGKSPNIVFADAELKKAVQNIILGIYYNSG
1bi9A          KIAFTGSTEVGKLIQEAAGRSNLKRVTLELGGKSPNIIFADADLDYAVEQAHQGVFFNQG
id. residues   K.AFTGST..G..I........LK.VTLELGGKSPNI.FADA.L..AV.....G...N.G

Model          EVCCAGSRVYVEESIYDKFIEEFKAASESIKVGDPFDESTFQGAQTSQMQLNKILKYVDI
1bi9A          QCCTAGSRIFVEESIYEEFVKRSVERAKRRIVGSPFDPTTEQGPQIDKKQYNKILELIQS
id. residues   ..C.AGSR..VEESIY..F...........VG.PFD..T.QG.Q....Q.NKIL.....

Model          GKNEGATLITGGERLGSKGYFIKPTVFGDVKEDMRIVKEEIFGPVVTVTKFKSADEVINM
1bi9A          GVAEGAKLECGGKGLGRKGFFIEPTVFSNVTDDMRIAKEEIFGPVQEILRFKTMDEVIER
id. residues   G..EGA.L..GG..LG.KG.FI.PTVF..V..DMRI.KEEIFGPV.....FK..DEVI..

Model          ANDSEYGLAAGIHTSNINTALKVADRVNAGTVWINTYNDFHHAVPFGGFNASGLGREMSV
1bi9A          ANNSDFGLVAAVFTNDINKALMVSSAMQAGTVWINC------Y--------GE-------
id. residues   AN.S..GL.A...T..IN.AL.V.....AGTVWIN.........................

Model          DALQNYLQVKAVRAKLD---
1bi9A          FGLREYSEVKTVTVKIPQKN
id. residues   ..L..Y..VK.V..K.....
```

Fig. S2. Amino acid sequence alignment of *Saccharomyces cerevisiae* aldehyde dehydrogenase Ald4p (residue range: 31 to 519) and *Rattus norvegicus* retinal aldehyde dehydrogenase type two. The 3D model of yeast Ald4p (P46367) shown in **Fig. 1** derived from a model prediction based on template "1bi9A", *R. norvegicus* retinal dehydrogenase type two (with 50% sequence identity). This alignment is replicated from www.proteinmodelprotal.org.

Biology Open • Supplementary information

```
P46367 ALDH4_YEAST      1    MFSRSTLCLKTSASSIGRLQLRYFSHLPMTVPIKLPNGLEYEQPTGLFINNKFVPSKQNK    60
P05091 ALDH2_HUMAN      1    -------MLRAAARFGPRLGRRLLS-AAATQAVPAPNQQPEVFCNQIFINNEWHDAVSRK    52
                                     *:::*    **  * :*    *  :  **       . :****::  : ..*

P46367 ALDH4_YEAST     61    TFEVINPSTEEEICHIYEGREDDVEEAVQAADRAFSNG-SWNGIDPIDRGKALYRLAELI    119
P05091 ALDH2_HUMAN     53    TFPTVNPSTGEVICQVAEGDKEDVDKAVKAARAAFQLGSPWRRMDASHRGRLLNRLADLI    112
                             ** .:**** * **:: ** ::**::**:**  **. *  *. :*  .**: * ***:**

P46367 ALDH4_YEAST    120    EQDKDVIASIETLDNGKAIS-SSRGDVDLVINYLKSSAGFADKIDGRMIDTGRTHFSYTK    178
P05091 ALDH2_HUMAN    113    ERDRTYLAALETLDNGKPYVISYLVDLDMVLKCLRYYAGWADKYHGKTIPIDGDFFSYTR    172
                             *:*:  :*::*******    *   *:*:*:: *:  **:*** .*: *  .  .****:

P46367 ALDH4_YEAST    179    RQPLGVCGQIIPWNFPLLMWAWKIAPALVTGNTVVLKTAESTPLSALYVSKYIPQAGIPP    238
P05091 ALDH2_HUMAN    173    HEPVGVCGQIIPWNFPLLMQAWKLGPALATGNVVVMKVAEQTPLTALYVANLIKEAGFPP    232
                             ::*:*************** ***:.***.***.**:*.**.***:****:: * :**:**

P46367 ALDH4_YEAST    239    GVINIVSGFGKIVGEAITNHPKIKKVAFTGSTATGRHIYQS-AAAGLKKVTLELGGKSPN    297
P05091 ALDH2_HUMAN    233    GVVNIVPGFGPTAGAAIASHEDVDKVAFTGSTEIGRVIQVAAGSSNLKRVTLELGGKSPN    292
                             **:*** ***  .* **:.* .:.********  ** *  : .::.**:***********

P46367 ALDH4_YEAST    298    IVFADAELKKAVQNIILGIYYNSGEVCCAGSRVYVEESIYDKFIEEFKAASESIKVGDPF    357
P05091 ALDH2_HUMAN    293    IIMSDADMDWAVEQAHFALFFNQGQCCCAGSRTFVQEDIYDEFVERSVARAKSRVVGNPF    352
                             *:::**::. **::  :.:::*.*: ******.:*:*.***:*:*.  * ::*  **:**

P46367 ALDH4_YEAST    358    DESTFQGAQTSQMQLNKILKYVDIGKNEGATLITGGERLGSKGYFIKPTVFGDVKEDMRI    417
P05091 ALDH2_HUMAN    353    DSKTEQGPQVDETQFKKILGYINTGKQEGAKLLCGGGIAADRGYFIQPTVFGDVQDGMTI    412
                             *..* ** *..: *::*** *:: **:***.*: **   ..:****:*******::.* *

P46367 ALDH4_YEAST    418    VKEEIFGPVVTVTKFKSADEVINMANDSEYGLAAGIHTSNINTALKVADRVNAGTVWINT    477
P05091 ALDH2_HUMAN    413    AKEEIFGPVMQILKFKTIEEVVGRANNSTYGLAAAVFTKDLDKANYLSQALQAGTVWVNC    472
                             .********: : ***: :**:. **:* *****.:.*.:::.*  ::: ::*****:*

P46367 ALDH4_YEAST    478    YNDFHHAVPFGGFNASGLGREMSVDALQNYLQVKAVRAKLDE---                   519
P05091 ALDH2_HUMAN    473    YDVFGAQSPFGGYKMSGSGRELGEYGLQAYTEVKTVTVKVPQKNS                   517
                             *: *    ****:: ** ***:.  .** * :**:* .*: :
```

Fig. S3. Amino acid sequence alignment of *Saccharomyces cerevisiae* aldehyde dehydrogenase Ald4p (P46467) and *Homo sapiens* aldehyde dehydrogenase ALDH2 (P05091). Their amino acid sequences are 48% identical (252 amino acids are identical and 148 amino acids are similar). The alignment was run using CLUSTALO available at www.uniprot.org. Mitochondrial transit sequence (1-24 for Ald4p, 1-17 for ALDH2) is highlighted in blue, transition state stabilizer residue (N192 for Ald4p, N186 for ALDH2) in purple, NAD-binding site (268-273 for Ald4p, 262-267 for ALDH2) in cyan, active site/proton acceptor (E290 for Ald4p, E285 for ALDH2) in red, and active site/nucleophile (C324 for Ald4p, C319 for ALDH2) also in red.

Table S1. List of primers used for recombinant DNA cloning, making DNA cassettes for yeast transformation, and DNA sequencing.

Primer Code	Sequence (5' to 3')	Description	Used with	PCR Product Size
**For site-directed mutagenesis of pFA6a-ALD4-GFP-kanMX6 (to introduce N192D, S269A, E290K, or C324A mutation into *ALD4* coding sequence) DNA template: pFA6a-ALD4-GFP-kanMX6**				
CN0025	5'-CAGATTATTCCTTGG**GAT**TTCCCACTGTTGATG -3'	Forward, with **N192D** mutation (in bold)	CN0026	6,434 bp
CN0026	5'- CCCACAAACACCCAAAGGCTG -3'	Reverse	CN0025	
CN0027	5'-GTTGCCTTCACAGGG**GCT**ACGGCTACGGGTAGAC -3'	Forward, with **S269A** mutation (in bold)	CN0028	6,434 bp
CN0028	5'- CTTTTTGATTTTTGGATGGTTTG -3'	Reverse	CN0027	
CN0029	5'-AAAAAAGTGACTTTG**AAA**CTGGGTGGTAAATCAC -3'	Forward, with **E190K** mutation (in bold)	CN0030	6,434 bp
CN0030	5'- CAAGCCTGCGGCTGCGGAC -3'	Reverse	CN0029	
CN0031	5'-AATTCTGGTGAGGTC**GCT**TGTGCGGGTTCAAGG -3'	Forward, with **S324A** mutation (in bold)	CN0032	6,434 bp
CN0032	5'- GTAGTAGATACCAAGGATAATGTTTTG -3'	Reverse	CN0031	
**For making DNA cassette carrying (5' to 3'): 50 nt upstream of *ALD4* start codon + *ALD4* coding sequence [*without nucleotides coding for MTS + desired mutation (N192D, S269A, E290K, or C324A)*] + GFP + kanamycin resistance gene + 50 nt downstream of *ALD4* stop codon DNA template: pFA6a-ALD4(N192D)-GFP-kanMX6, pFA6a-ALD4(S269A)-GFP-kanMX6, pFA6a-ALD4(E290K)-GFP-kanMX6, pFA6a-ALD4(C324A)-GFP-kanMX6**				
CN0003	5'-GTATCTGGAAAACCAACCAAGAAAACTACAATAACAAAAATAAATAAAGC**ATG**TCACACCTTCCTATGACAGTG -3'	Forward, with 50 nt upstream of ***ALD4*** start codon, **nt73** (underlined) is placed right after start codon (in bold).	CN0004	4,012 bp
CN0004	5'-TTAATTTTATGTATGTAAGCATCGATTGGACACCAGGCTTATTGATGACCATCGATGAATTCGAGCTCG -3'	Reverse, with 50 nt downstream of ***ALD4*** stop codon, sequence homology to pFA6a-ALD4-GFP-kanMX6 (underlined).	CN0003	
**For making DNA cassette carrying (5' to 3'): *ALD4* coding sequence [*with nucleotides coding for MTS*] + GFP + kanamycin resistance gene + 50 nt downstream of *ALD4* stop codon DNA template: pFA6a-ALD4-GFP-kanMX6**				
CN0047	5'- **ATG**TTCAGTAGATCTACGCTCTG -3'	Forward; nt 1-23 of ***ALD4*** coding sequence, start codon (in bold).	CN0004	4,031 bp
CN0004	5'-TTAATTTTATGTATGTAAGCATCGATTGGACACCAGGCTTATTGATGACCATCGATGAATTCGAGCTCG -3'	Reverse, with 50 nt downstream of ***ALD4*** stop codon, sequence homology to pFA6a-ALD4-GFP-kanMX6 (underlined).	CN0047	
**For PCR verification of yeast transformants (to check if MTS is removed from *ALD4*, in the yeast genome)**				
CN0009	5'- GGAAAATGAGGGGCGGGTGTAG -3'	Forward; 200 nt upstream of ***ALD4*** start codon.	JW1623	2670 bp (having MTS), 2601 bp (MTS removed)
JW1623	5'- GCGACCTCATACTATACCTG -3'	Reverse; 164 nt downstream of **GFP** stop codon.	CN0009	
**For verification of MTS removal together with N192D, S269A, E290K, or C324A mutation in genomic *ALD4* by DNA sequencing**				

**Primer Code**	**Sequence (5' to 3')**	**Description**	**Used with**	**PCR Product Size**
CN0009	5'- GGAAAATGAGGGGCGGGTGTAG -3'	Forward; 200 nt upstream of ***ALD4*** start codon		
CN0010	5'- AAGATTGCCCCTGCTTTGG -3'	Forward; nt 601-619 of ***ALD4*** coding sequence		
CN0011	5'- TACTTCATTAAGCCAACTGTCTTTG -3'	Forward; nt 1201-1225 of ***ALD4*** coding sequence		

Biology Open • Supplementary information

Table S2. Data, used to plot graph in Fig. 1, showing percentage of yeast cells *ALD4(noMTS)::GFP* with Ald4p(noMTS)-GFP structures after treated with acetaldehyde or sterile water for 15 min.

Experiment #	Percentage of cells with Ald4p(noMTS)-GFP asssembly			
	Clone 1		Clone 2	
	+Acetaldehyde	+Sterile water	+Acetaldehyde	+Sterile water
1	98.05	1.56	98.43	1.49
2	98.48	1.15	98.83	1.89
3	98.86	1.52	99.23	1.95
**Average**	**98.46**	**1.41**	**98.83**	**1.78**
**SEM**	**0.234**	**0.131**	**0.231**	**0.144**
**P-value (two-tailed)**	**<0.0001******		**<0.0001******	
